# Development of Pavement Material Using Crumb Rubber Modifier and Graphite Nanoplatelet for Pellet Asphalt Production

**DOI:** 10.3390/polym15030727

**Published:** 2023-01-31

**Authors:** Jong-Sub Lee, Sang-Yum Lee, Yoon-Shin Bae, Tri Ho Minh Le

**Affiliations:** 1 National Research Council Research Associate, Turner-Fairbank Highway Research Center, Federal Highway Administration, 6300 Georgetown Pike, F211, Mclean, VA 22101-2296, USA; 2 Faculty of Civil Engineering, Induk University, 12 Choansan-ro, Nowon-gu, Seoul 01878, Republic of Korea; 3 Department of Safety and Disaster Prevention Research, 37 Maebongsan-ro, Mapo-gu, Seoul 03909, Republic of Korea; 4 Faculty of Civil Engineering, Nguyen Tat Thanh University, 300A Nguyen Tat Thanh Street, District 4, Ho Chi Minh City 70000, Vietnam

**Keywords:** crumb rubber modifier, graphite nanoplatelet, pellet asphalt, pothole restoration, recycling byproduct

## Abstract

The purpose of this research was to promote the recycling of pellet asphalt with Crumb Rubber Modifier (CRM) and Graphite Nanoplatelet (GNP) in pothole restoration. In this study, several laboratory tests were carried out on mixes containing CRM content ratios of 5%, 10%, and 20% and GNP content of 3% and 6% in order to identify the ideal mixing ratio of pellet-type asphalt paving materials. The Marshall stability test, the Hamburg wheel tracking test, and the dynamic modulus test were all performed to compare the effectiveness of the proposed method and heated asphalt combinations. Afterward, the full-scale testbed was conducted to verify the practical application between the proposed method and popular pothole-repairing materials. Both laboratory and field test findings confirmed that the asphalt pavement using 5% CRM and 6% GNP improved the resistance to plastic deformation and anti-stripping compared to the generally heated asphalt paving material, thereby extending road life. However, the resistance to fatigue cracking can be slightly reduced by incorporating these additives. Overall, the CRM and GNP asphalt pellet approach is a feasible solution for sustainable pavement maintenance and rehabilitation, particularly in small-scale damage areas such as potholes.

## 1. Introduction

Small-scale pavement damage is rapidly increasing due to environmental impacts and increased traffic volume [1,2,3]. The pavement distress can also be attributable to improper material characteristics, inappropriate mix design features, and poor construction works [4]. The presence of this minor pavement damage can lead to the progressive development of a higher degree of pavement deterioration and the potential for traffic accidents. When these small road damages (i.e., potholes) and urgent repairs are required, emergency restoration must be performed abruptly to prevent traffic problems [5,6]. Currently, conventional heated asphalt concrete is difficult to produce in small quantities and the plant operation must be stopped in the case of rain. Reports indicated that construction defects and quality deterioration occur due to the temperature drop of the asphalt mixture when conventional heated liquid asphalt concrete is transported and stored at the site [7,8]. Therefore, in order to solve this problem, if the asphalt mixture is made into a solid material (pellet), it is easy to transport and store, so it can be used for emergency restoration work by using small-scale heating equipment at the emergency repair site after excavation restoration that requires construction [9,10]. Moreover, every year in summer and winter, the number of potholes increases rapidly and causes a lot of damage. Since potholes occur steadily every year and threaten the safety of drivers, it is necessary to develop pothole recovery construction technology and high-durability pavement materials [11]. Currently, room-temperature pellet asphalt is applied to emergency restoration work, but there is a problem in that durability has remained at low quality. 

According to numerous surveys, approximately 2 billion discarded tires are eliminated globally annually [12]. This makes up between two and three percent of the total amount of gathered garbage. About 300 million used tires come to trash each year in the US alone [12]. Global population growth is accompanied by an increase in automobile consumption, particularly among the growing middle classes in developing nations that have more access to automobiles [13]. Since more pavement miles are traveled, more tires need to be substituted, which results in more waste tires [14]. Waste tires are a recent threat that causes a big concern to the planet. Rubber is still disposed of incorrectly regardless of recycling activities and restrictions of authority [15]. Tires potentially leak pollutants into the air, soil, and groundwater that can disrupt the environment as they accumulate in landfills [16]. A discarded tire emits methane gas into the atmosphere just by being exposed to the sunlight. The carbon emissions grow as a result of this greenhouse gas, which may also lead to global warming.

Recycling waste tires is proving to be a growing practical solution to the world’s waste tire issue. While just a small portion of discarded tires are recovered annually, recycling has become the main strategy for waste tire treatment in Europe, where it accounts for 40% of all discarded tires [17]. Therefore, the practical recycling of waste tires on a large scale would significantly promote sustainable development purposes [18]. 

Hundreds of thousands of tons of waste tires annually can be turned into waste globally [19,20]. In Korea, the recycling rate of domestically recycled tires was 292,434 tons or about 79% of the annual amount of waste tires. Therefore, it is necessary to strengthen the recycling method of high-value-added materials. The Korean Ministry of Land, Transport, and Maritime Affairs has been enforcing the recycling of industrial byproducts such as waste tires into road pavement in recent years. The purpose was to activate the recycling of waste tire powder in general road pavement, as it is currently limited in use for playground and pedestrian roads. Recycling industrial by-products reduces damage such as plastic deformation of asphalt and concrete road pavements and increases durability, increasing road life [21,22,23,24]. Mixing waste tire rubber powder increases plastic deformation resistance by 4.6 times, which is effective in long-term service life reinforcement. The vitalization of this application is expected to reduce the cost of asphalt concrete materials while expanding eco-friendly recycling purposes [20].

Waste tire rubber powder has been used as a binder for heated asphalt mixtures since 1975 and has been used as a binder for various types of heated asphalt mixtures until now. Crumb Rubber Modifier (CRM) is a general term for rubber powder of waste tires. CRM asphalt was initially used for crack patching called Band-Aid, and it was applied to the chip-seal method while mechanized spraying was possible and used for repairing cracked roads [25,26]. The advantages of CRM asphalt are its high strength, resistance to plastic deformation, reducing environmental pollution problems [27,28]. Crumb Rubber Modified Asphalt (CRM asphalt) containing waste tire rubber has higher viscosity at high temperatures and lower stiffness at low temperatures than general asphalt. High-viscosity asphalt binder has high resistance to high stress and deformation caused by traffic load [29,30,31], and low stiffness at low temperatures has high resistance to low-temperature cracking. Additionally, the storage stability tests in the research of H. Kim and S.J. Lee indicated that the viscosity of the CRMAs had a significant influence on the phase separation of the binders; the higher the viscosity of the binder, the less the degree of the separation [18]. In addition, due to the elasticity of the rubber component, it has a good effect on reflective cracking, and the carbon black present in waste tires has an effect of preventing asphalt oxidation [32,33,34]. The crack resistance and plastic deformation resistance characteristics of CRM asphalt can compensate for the problem of deterioration in the durability of room-temperature asphalt concrete used during emergency restoration work [22]. 

However, when the single construction area is small and the construction period is short, additional costs such as frequent movement of production facilities, installation, disassembly, and storage of pavement materials (temperature maintenance) are incurred. In addition, in the Korean national pavement survey after road excavation and compaction, the overall flatness of pavement is acceptable, but structurally quality is confirmed at a poor level since the crack rate and plastic deformation occur above a certain level which has required continuous repairing budget. This results in an increasing need for practical overlaying materials which can ensure the strength of the asphalt surface layer. In order to solve this problem, an asphalt mixture made into a pellet and small-scale heating equipment is used for emergency pavement restoration work such as potholes. 

**Figure 1:**
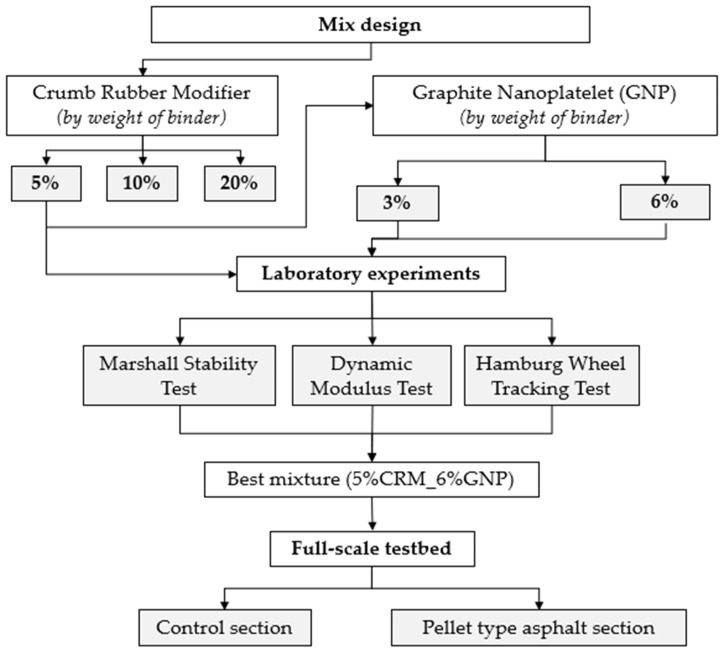
Testing flowchart.

In this research, in order to determine the optimal mixing ratio of pellet-type asphalt paving materials having recycled waste tire powder, various laboratory tests were conducted on mixtures with CRM content ratios of 5%, 10%, and 20%. The generally heated asphalt mixtures were incorporated to compare the performance between the developed and conventional solutions. For performance evaluation, the Marshall stability test, Hamburg wheel tracking test, and dynamic modulus test were conducted. Additionally, for the purpose of improving the performance of asphalt paving materials, the same test was performed on asphalt paving materials GNP (Graphite Nano Platelet, 3% and 6%). To compare and evaluate the performance with popular pothole repairing materials, the proposed CRM_GNP pellet asphalt pavement material was applied in the fields where potholes usually occur. The testing process was summarized in Figure 1. 

## 2. Materials and Methods

### 2.1. Materials

#### 2.1.1. Aggregates and Asphalt Binder

The Korean Plant, which is close to the Seoul metropolitan region, supplied the natural aggregate and fillers for this study. Table 1 displayed the aggregate’s fundamental characteristics. This study created specimens in line with the Superpave compaction method. The optimal asphalt binder makes up 6–7% of the combined mass, according to trial experiments and recommendations from related research [35,36,37]. 

The asphalt binders used in this work were upgraded with styrene-butadiene-styrene (SBS) [43]. SBS is widely acknowledged for its adaptability at low temperatures and its capacity to increase a binder’s flexibility at high temperatures [44]. By reducing the blending heat and the viscosity of high-temperature asphalt, this SBS modifier can improve the high efficiency of an asphalt mix and boost the adaptability of asphalt [45]. The designed asphalt is more resistant to rutting at higher temperatures as well as low-temperature cracking than regular asphalt. In South Korea, this material has a proven record of successful application [46]. Table 2 presents the general properties of asphalt modified with CRM by the weight of the binder.

Regards to the CRM, the mechanochemical approach was employed to generate the CRM from activated rubber powder with a particle size of 30 mesh. In order to facilitate the chemical reaction, a chemical activator (organic disulfide, or OD) was introduced with mechanical energy [22]. The activating procedure settings of the mechanochemical approach utilizing the OD additive (3% of crumb rubber powder by weight) were adjusted following the prior research [30]. The initial laboratory suggested that the mixing of SBS, tire, and GNP leads to very high viscosity. Therefore, the mixing and compaction temperature were controlled at a quite high degree to ensure a homogenous mixture and acquire a proper air void level. The mixing process was controlled at 165 °C for 30 min with a 3% OD additive concentration while the compaction was designed at 155 °C for the compaction process, based on the mixing and compaction guiding from Al Mamun et al. [47].

**Table 2:** Properties of asphalt binder.

Properties	Value	Standard Value
Penetration (1/10 mm) 25 °C [48]	86.1	
Softening point (°C) [49]	69.2	
Ductility at 5 °C (cm/min) [50]	103	
Thin film oven (160 °C, 300 min) [51]		
Mass loss (%) [51]	0.03	
Penetration loss [51]	69	
G*/sinδ; at 76 °C (Original) [52]	1.68 kPa	Min. 1.0 kPa
G*/sinδ at 76 °C (after RTFO) [52]	2.39 kPa	Min. 2.2 kPa
G* × sinδ at 76 °C (after PAV) [53]	1498 kPa	Max. 5000 kPa
Stiffness at −22 °C [54]	176 MPa	Max. 300 MPa
m-value at −22 °C [54]	0.33	Min. 0.3

#### 2.1.2. Mixture Design

Mixture design is based on the particle size of the maximum aggregate particle size of 13 mm (WC-2), which is used as a standard, and three types of mixes of 5%, 10%, and 20% of rubber content were suggested based on the asphalt content of 6.0–7.0%. Table 3 shows the aggregate content of each sieve. A Graphite Nano Platelet (GNP) was introduced to the bitumen binder in different amounts: 3% and 6% by mass of the binder. This GNP is composed of synthesized graphite substance with 99.58% carbon and 0.42% ash, and it has an optimum surface area of 245 m^2^/g [55]. In general, the CRM and GNP types used in this research were very popular and they can be provided by manufacturers worldwide [30,55]. In this study, GNP was added to improve the performance of CRM asphalt paving materials, and Marshall stability, residual stability, wheel tracking tests, Hamburg wheel tracking, and dynamic modulus tests were performed to evaluate the performance of the CRM_GNP mixtures. 

When designing the mixture, the weight of the total mixture was based on 1000 g, and AP-5 was used as the asphalt binder used in the design of the mixture. Table 4 below shows the calculation results required for calculating the amount of material and designing the mixture.

### 2.2. Testing Methods

#### 2.2.1. Marshall Stability and Residual Stability Tests

The Marshall stability test is a test performed to determine the mixing in the process of manufacturing a heated asphalt mixture. ASTM D6927 [56] is regulating Marshall stability for flow values. This Marshall stability is used in mixing design by measuring the compaction stiffness of the asphalt mixture. For the purpose of determining the mixture for the target asphalt mixture, a load is applied at a constant speed of 50.8 mm/min, and the maximum load is measured when the flow value is between specific values. For the maximum load, a correction factor is applied according to the test specimen height of the mixture. Residual stability is a factor that evaluates the stripping resistance of an asphalt mixture and can be obtained by the ratio of the initial mixture and the mixture immersed at 60 °C for 48 h.

(1) $$\mathrm{Residual}\;\mathrm{stability}=\frac{\mathrm{Stability}\;\mathrm{after}\;48\;\mathrm{hours}\;\mathrm{of}\;\mathrm{water}\;\mathrm{immersion}\left(N\right)}{\mathrm{First}\;\mathrm{Stability}\left(N\right)}$$

#### 2.2.2. Wheel Tracking and Hamburg Wheel Tracking Test

**Figure 2:**
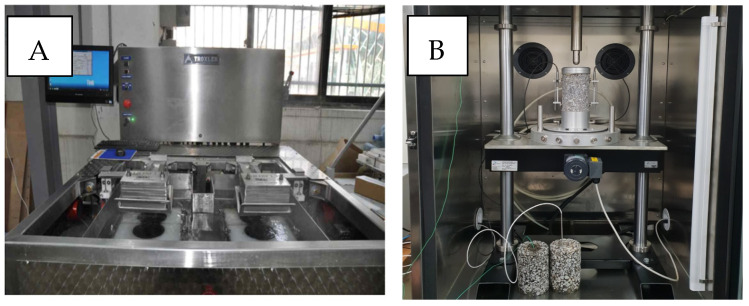
(**A**) Hamburg wheel tracking test and (**B**) dynamic modulus test.

The wheel tracking test is a test method that determines the dynamic stability by repeatedly loading a load indoors to evaluate the plastic deformation characteristics of an asphalt mixture due to a vehicle load at a high temperature. Dynamic stability indicates the resistance to plastic deformation. The lower the dynamic stability, the greater the depth of settlement, and the higher the dynamic stability, the smaller the settlement. This is regulated in AASHTO 324 [57], and the frequency of the test varies depending on the type of pavement of the asphalt mixture. The specimen is made into a rectangular parallelepiped and fixed to the tester as shown in Figure 2A. The traffic load is generally applied at 686 N, realizing a situation where vehicles pass at 42 times/minute. The test is evaluated by the number of times the wheel passes with respect to the unit deformation amount of 15 min intervals between the time point after 45 min and the time point of 60 min. This is called the strain rate, and the general strain rate and dynamic stability can be obtained as follows.

(2) $$\mathrm{Strain}\;\mathrm{rate}\;\left(\mathrm{RD},\;\mathrm{mm}/\min\right)=\frac{d_{60}-d_{45}}{15}$$

(3) $$\mathrm{Dynamic}\;\mathrm{stability}\;\left(\mathrm{DS}\right)=\frac{42\times c\times \left(t_{2}-t_{1}\right)}{\left(d_{2}-d_{1}\right)}=\frac{42\times 1\times \left(60\min-45\min\right)}{\left(d_{60\min}-d_{45\min}\right)}$$

The Hamburg wheel tracking test is similar to the wheel tracking test, but it is a test method by immersion in water. The size of the specimen is different and the moisture sensitivity can be evaluated. In this test, the moisture sensitivity is determined by considering the point where the amount of settlement rapidly changes as the point of stripping.

#### 2.2.3. Dynamic Modulus Test

The dynamic modulus test is performed by attaching a strain measurement sensor (LVDE, Linear Variable Differential Transformer) to the specimen, measuring temperature (5, 20, 40 and 54 °C) and load cycle (0.1, 0.5, 1.0, 5.0, 10 and 20 Hz) to measure the dynamic modulus, phase angle, and deformation [58]. It is a test method to describe the actual road environment considering the temperature and vehicle traffic according to the season (see Figure 2B). The master curve can be drawn by applying the Time-Temperature Superposition Principle (TTSP), and the final master curve is drawn by shifting the dynamic elastic coefficient of the frequency band according to the temperature and frequency. The function expression for this is:

(4) $$\log\left|a_{T}\right|=\delta +\frac{C_{1}\left(T-T_{d}\right)}{C_{2}+\left(T-T_{ref}\right)}$$

where *C*_1_ and *C*_2_ = the regression parameters, *T* = the measured temperature, *T_d_* = the defined temperature; and *T_ref_* = the reference temperature (i.e., 20 °C).

The dynamic modulus test is a concept that can evaluate the degree of physical resistance to Visco-Elastic materials. The dynamic modulus is the most common test that can represent the performance of asphalt mixtures and is performed according to the AASHTO TP 62 standard.

### 2.3. Introduction of Pellet-Type Asphalt Technology

#### 2.3.1. Conventional Liquid Heating Technology

**Figure 3:**
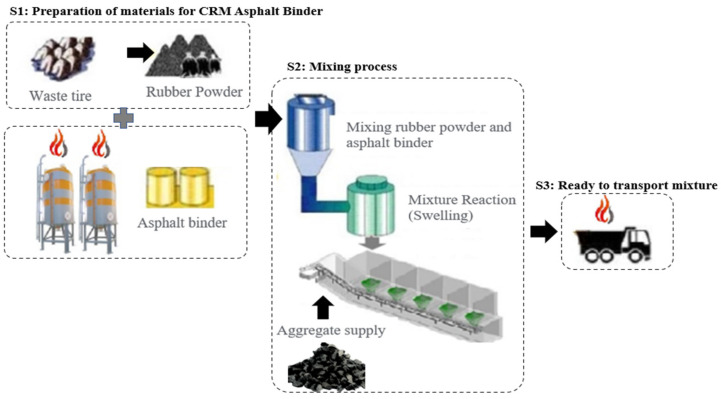
CRM asphalt pavement manufacturing process.

The conventional mixing method is a method of producing asphalt mixture pavement materials by mixing aggregate with CRM asphalt, which is a mixture of fine waste tire rubber powder and asphalt at about 210 ± 10 °C. Wet production technology includes batch technology in which 15 to 20% of CRM powder is added as a polymerization ratio to the asphalt and reacted at 162 to 190 °C and the continuous blending technology. The production technology commonly used in Korea requires a swelling process of CRM powder (see Figure 3). Through this, it is possible to increase the bonding strength between the rubber powder and the asphalt surface while maintaining the elastic properties of rubber. To this end, a large set of equipment, namely a heat and blender and a reaction tank that stores CRM asphalt is essential. 

#### 2.3.2. Pellet Asphalt Technology

**Figure 4:**
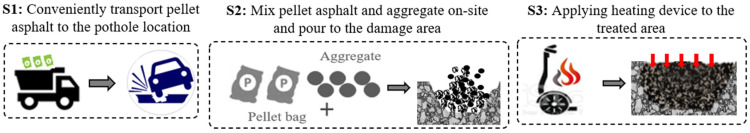
Pellet-type asphalt pavement is applied to the emergency pothole repair site.

When the asphalt mixture is made into a solid material (pellet), it is easy to transport and store, so it can be used for emergency restoration work by using small-scale heating equipment at the emergency repair site after excavation restoration that requires construction. There are different methods for producing asphalt pellets, including extrusion techniques utilized in the polymerization industry. Figure 4 summarizes the application of pellet-type asphalt pavement. 

This study initiates the production process by synthesizing the binder to the correct grade and adding the proper content of modifiers. The modified binder is combined with the recommended solid additives when it satisfies all of the specification requirements, and it is then pelletized. In order to avoid clumping during transportation and handling, a non-stick covering is sprayed to coat the pellet. 

Initially, the binder pellets’ cores were formed by blending asphalt binder with 5% CRM and 3% GNP. Three (3) min of mixing at 165 °C and 1500 rpm were sufficient to guarantee uniform dispersion. The pellets are then encapsulated in a protecting coating in the following phase. For that, a glass container was filled with 1000 mL of distilled water and 30 g of alginate. A uniform emulsion was produced by mixing the two ingredients in a mixer at 500 rpm for 12 min, using the alginate to function as the surfactant. In a different glass container, 1000 mL of distilled water was combined with 30 g of calcium chloride to create a calcium chloride mixture. The cores were initially transferred to the alginate emulsion, then to the calcium chloride mixture, enabling the alginate to ionotropic gel on the pellets’ entire exterior. 

**Figure 5:**
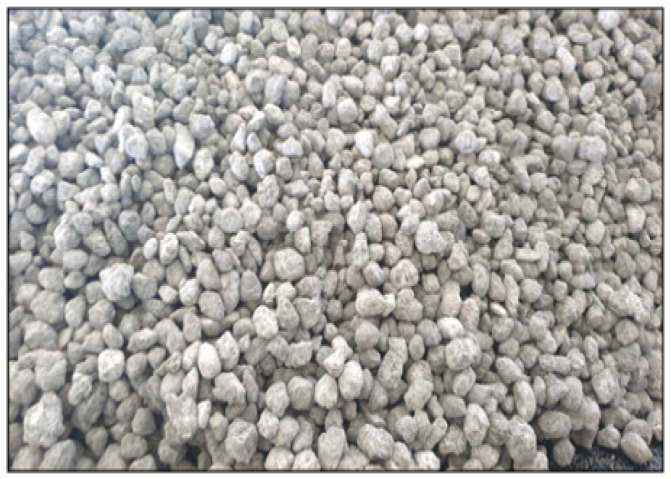
The manufacturing process of pellet-type asphalt pavement and pictures of paving materials.

The mixture was then cured for 6 h at 3 °C to create the pellets in Figure 4. It is possible to stabilize the pellets’ internal structure by using an alternative cooling fan. Afterward, pellets were kept in room condition in polyethylene containers until construction day. Figure 5 shows the photograph of the manufactured pavement material.

### 2.4. Field Application

#### 2.4.1. Field Pothole Restoration 

Based on the performance evaluation analysis results in the previous section, in order to apply CRM (5%) asphalt paving materials to emergency repair work, constructability and commonality are confirmed through construction and monitoring at sites where potholes frequently occur.

**Figure 6:**
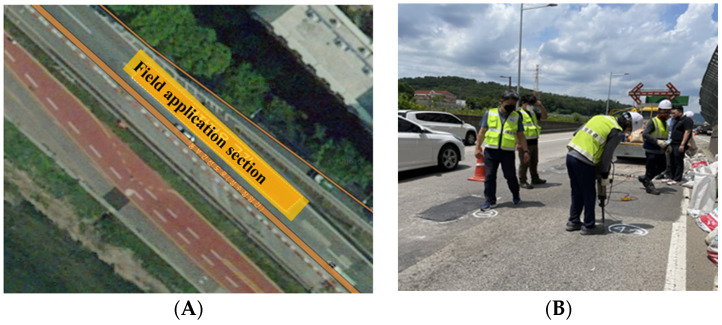
(**A**) Field application location and (**B**) view of the construction site.

Test construction was carried out on 14 July 2021, on the Dongbu Arterial Road located in Nowon-gu with the cooperation of the Seoul Facilities Corporation. Three types of existing pothole repair agents (medium heating type, water hardening, general room temperature asphalt concrete) were used and built simultaneously. Figure 6 shows a schematic diagram of the site application location and a panoramic view of the construction site.

**Figure 7:**
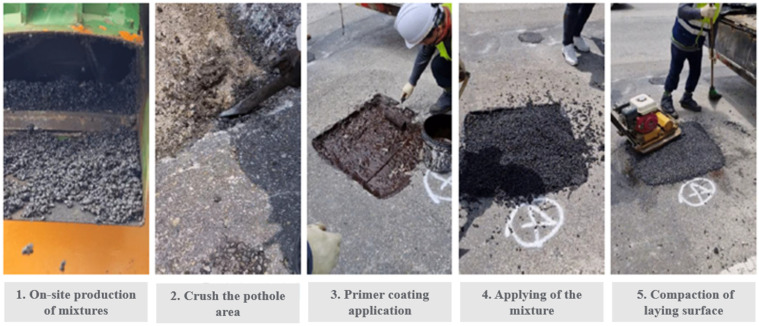
Step-by-step construction process.

In the construction process, the section where the existing pothole occurred is crushed using a hand crusher, primer is applied to the crushed surface to improve adhesion with the paving material, paving material is laid on the surface of the primer and compacted using a vibrating rammer. Figure 7 below shows the step-by-step construction process.

A total of 3 types of CRM (5%) pavement material mixtures were selected for comparison. Medium-temperature type repair materials, hydraulic repair materials, and normal room temperature repair materials, which are most commonly used in pothole repair sites in Seoul, were selected. 

#### 2.4.2. Falling Weight Deflectometer Test

**Figure 8:**
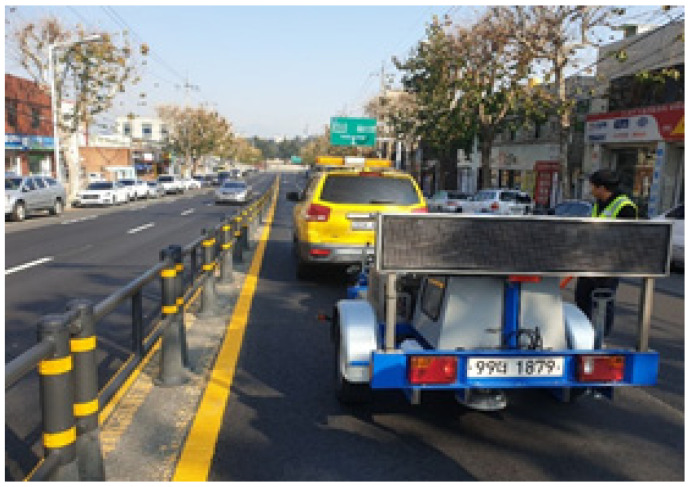
FWD test.

This research employed a Falling Weight Deflectometer (FWD) test to assess the structural capacity following the execution of the field experiment, as illustrated in Figure 8. The research was carried out to evaluate each section’s structural attributes. By exposing the pavement surface to an applied load and measuring the surface displacement using a variety of instruments that have been mounted on the pavement surface, the FWD test is a non-destructive testing device developed to determine the structural characteristics of pavement constructions. The size of the load plate is 30 cm, and the typical load is 4082 kgf. The geophones monitor the displacement at each point once a dynamic load is imposed [59,60,61].

**Figure 9:**
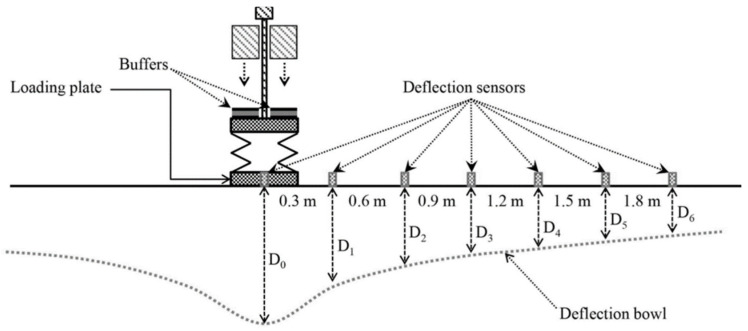
FWD deflection bowl with measuring geophone set-up.

The obtained FWD displacements, Dr, at geophones placed at different distances, r in mm, from the center of pressure are used to determine the displacement bowl. The typical FWD test geophone configuration is positioned at D0 (under the center of the FWD loading frame), 200, 300, 450, 600, 900, 1200, 1500, and 1800 mm, respectively [62]. In these settings, displacements caused by falling weights, such as 4082 kgf, are monitored. Figure 9 shows these separate monitoring sites.

The deflection bowl’s separate measurement sites allow for the determination of deflection variables that describe specific zones or regions of the entire deflection bowl [63,64,65]. For each form of asphalt pavement mix utilized in this investigation, the FWD test was conducted at 20 m spacing. When the actual test was conducted in Nowon-gu, South Korea, the air temperature was around 8 °C, and the asphalt pavement layer’s average temperature was around 9 °C.

#### 2.4.3. Falling Weight Deflectometer (FWD) Analysis

The most fundamental formulae for evaluating pavement structure were applied in this research to examine the pavement structure [65,66,67,68]. Additionally, the structural condition rating was divided in accordance with Table 5 [63] to assess it. The displacement in the middle of the loading plate (D_0_) and the AREA, which is an examination of the displacement basin features, are the FWD parameters that are most commonly utilized to determine the structural integrity of the pavement. The AREA can be calculated using Equation (5).

(5) $$\mathrm{AREA}=6(1+2\frac{D_{300}}{D_{0}}+\frac{D_{600}}{D_{0}}+\frac{D_{900}}{D_{0}})$$

where D_0_ is the deflection in the center of the load plate (in μm), D_300_, D_600_, and D_900_ are the deflection (μm) at 300, 600, and 900 mm from the core of the loading plate.

Surface curvature index (SCI) assesses the stability of the surface layer, base curvature index (BCI) the base layer, and base damage index (BDI) the road for the sub-base. The deviation increases as each index’s value increases. Each index’s calculation is demonstrated in Equations (6)–(8).

(6) $$\mathrm{SCI}=D_{0}-D_{300}$$

(7) $$\mathrm{BDI}=D_{300}-D_{600}$$

(8) $$\mathrm{BCI}=D_{600}+D_{900}\;$$

RoC (radius of curvature) is an index indicating the structural state of the surface and base layers, and is calculated by the following Equation (9):

(9) $$\mathrm{RoC}=\frac{L^{2}}{2D_{0}(1-\frac{D_{200}}{D_{0}})}$$

where L: 200 (mm), and $D_{200}$: deflection at a distance of 200 mm from the center of the load plate (μm).

Additionally, a Modulus 6.0 inverse analysis was also carried out. The displacement based on the multilayer elastic theory was calculated employing the inverse approach employing the size of every layer and the estimated elastic modulus range of each layer. The elastic modulus for every layer was changed, and the computations were performed until the calculated deflection fitted the values of the measurements. This method allows for the exact estimation of each layer’s elastic modulus.

## 3. Results

### 3.1. Derivation of Optimum Asphalt Mixing Ratio for Each CRM Content

Marshall stability results of each asphalt mixture are shown in Table 6. The optimal asphalt mixing test was conducted three times and the average value was used. The optimal asphalt mixing ratio was 6.0% at 5% CRM content while the optimal asphalt mixing ratio was 6.5% at a CRM of 10%. Considering the asphalt at 20% CRM content, the optimal blending ratio was found to be 7.0%. However, in the case of the CRM content of 20%, the porosity was 2.6%, which was below the standard, and the flow value was 40, which was above the standard. These test results also reveal that CRM content at 20% also leads to a noticeable drop in Marshall stability which may be inappropriate for pothole restoration purposes. Therefore, the CRM contents that satisfied the basic properties were 5% and 10%. Through this phase, based on the superior behavior of the 5% CRM mixture, the CRM was fixed at this lowest level in the following test to ensure the strength of the asphalt concrete mixture while promoting sustainability purpose. 

### 3.2. Performance Comparison of Asphalt Paving Materials

#### 3.2.1. Marshall Stability and Residual Stability

**Figure 10:**
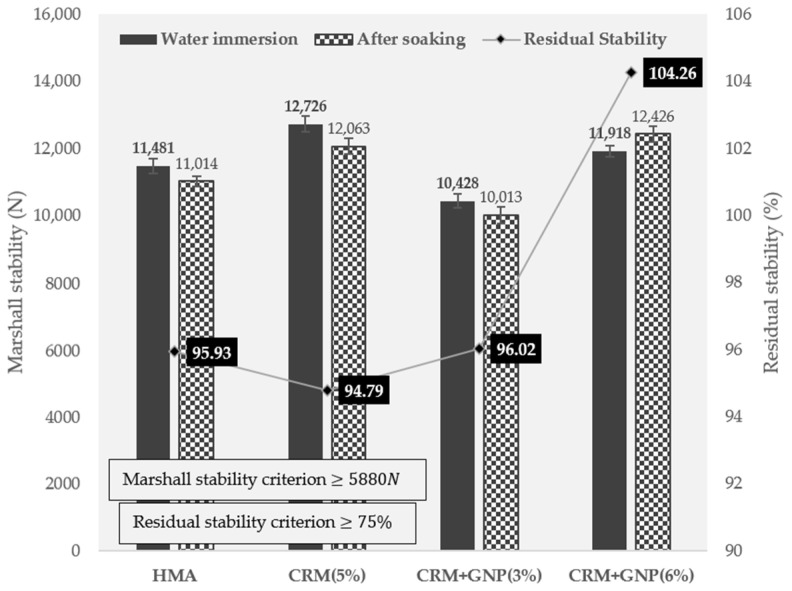
Marshall stability test results.

Figure 10 shows the Marshall stability results of heated asphalt concrete mixture (HACM) and CRM (5%) asphalt mixture. The results of adding GNP (3% and 6%) into the 5% CRM mixture were also incorporated. In general, the Marshall stability values of modified mixtures show similar results compared to the reference mix, and a significant effect on plastic flow resistance was not found in these conditions. The test results present that the Marshall stability of the reference, 5% CRM, 5% CRM + 3% GNP, and 5% CRM + 6% GNP mixtures are 11,481, 12,726, 10,428, and 11,918 N, respectively. The CRM asphalt mixture (5%) showed a slightly lower residual stability than the heated asphalt mixture (HMA), but the addition of GNP (3% and 6%) confirmed the increased residual stability and improved the resistance to anti-stripping. For example, the addition of 3% and 6% GNP leads to the residual stability increase to 96.02 and 104.26%, respectively, while the value of the CRM 5% mixture was 94.79%, noticing an improvement in water resistance effectiveness. This can be attributed to the dense structure of HMA having proper content of GNP which results in the stronger moisture stability of the modified mixture. 

#### 3.2.2. Wheel Tracking and Hamburg Wheel Tracking Test

**Figure 11:**
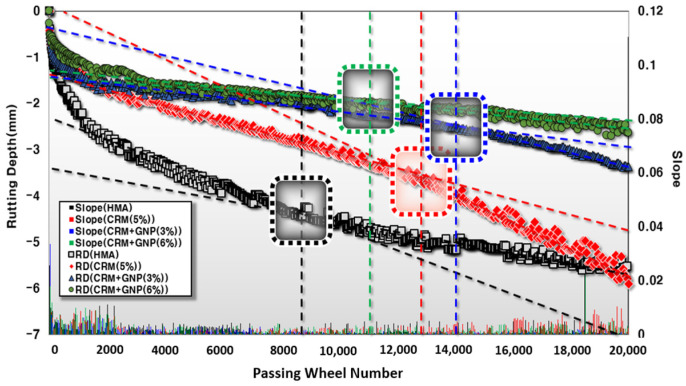
Hamburg wheel tracking test results.

**Figure 12:**
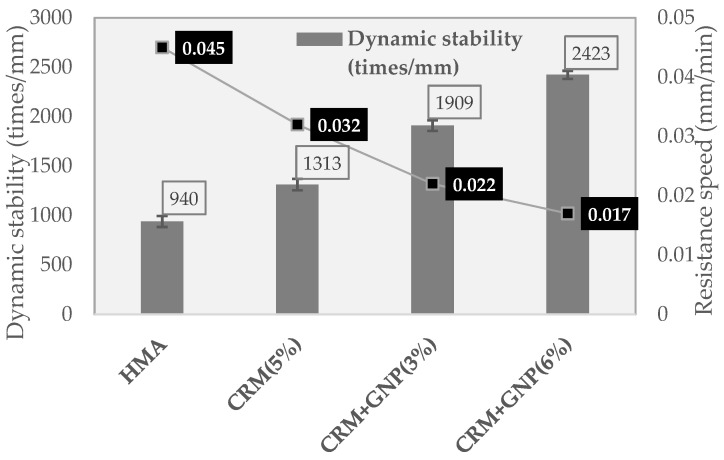
Dynamic stability based on the HWT test.

Figure 11 demonstrates the HWT test results among all mixtures. The test results reveal that the reference mixture suffered from a noticeable increase in the rutting value compared to the modified mixture having CRM and GNP. By analyzing the rutting depth (mm) value when the wheel load reaches 20,000 times, the HWT test shows that the GNP additive is effective in reducing plastic deformation. For example, in the case of the mixture of HMA and CRM (5%), about 6 mm of plastic deformation occurred. However, the plastic deformation in the mixture of CRM (5%) + GNP (6%) is about 2.1 mm, while the plastic deformation of about 3.3 mm was recorded in the mixture of CRM (5%) + GNP (3%). As shown in Figure 12, the dynamic stability (2423 times/mm) of 5% CRM + 6% GNP mixtures are significantly stronger than the dynamic stability of the 5% CRM mixture (1313 times/mm). This finding confirmed that the resistance to plastic deformation is remarkably improved (about 1.8 times higher). In addition, since it is a water immersion test, it is judged that the moisture resistance is also improved by adding GNP. This can be explained by the compacted structure of GNP modified mixture since moisture impact may be remarkably resisted. As a result, asphalt pavement materials corresponding to CRM (5%) + GNP (3%) showed the highest resistance to stripping. The case with the highest mixing ratio of GNPs (6%) shows vulnerability to exfoliation impact.

#### 3.2.3. Dynamic Modulus Test

**Figure 13:**
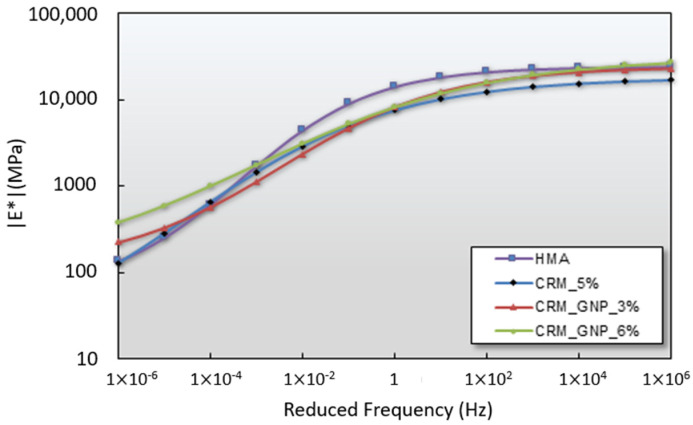
Dynamic modulus test results.

The results of the dynamic modulus test performed in this study are presented in Figure 13, and each axis of the graph is represented in a log scale. It is defined as the stress-dependent modulus of elasticity that changes according to the degree of the cyclic load and confining stress. The test results reflect the possibility of fatigue cracking of the pavement material under the condition that the pavement is subjected to repeated wheel loads due to vehicle driving. The higher the dynamic modulus, the lower the possibility of fatigue cracking. Based on the findings, the dynamic modulus of the reference HMA mixture slightly outperformed the modified mix. Considering multiple frequency ranges, the dynamic modulus of CRM asphalt pavement material was found to be equal to or lower than that of the heated asphalt pavement material (HMA). 

However, in the case of a CRM mixture modified by GNP 6%, the test results revealed that the dynamic modulus increases substantially at high temperature (low-frequency region) and low temperature (high-frequency region), indicating a remarkably improved resistance to plastic deformation and proper stability in the cold area. Therefore, this finding confirms that the 5% CRM+6% GNP mixture can be applied in pellet asphalt for pothole restoration in multiple regions since this condition shared the equivalent performance compared to the control mixture while promoting the environmentally friendly purpose. 

### 3.3. Performance Evaluation Analysis

In this study, a value comparison analysis was conducted on the tests performed. In detail, plastic flow resistance and stripping resistance by Marshall compaction test, plastic deformation and fatigue crack resistance were evaluated by the Hamburg wheel tracking test and dynamic modulus test, respectively. Although stripping resistance can be derived from the Marshall compaction test and the Hamburg wheel tracking test, the Marshall compaction test shows the anti-stripping of pure specimens, and the wheel tracking shows the stripping property in road driving conditions, so they were all classified and evaluated. On the other hand, the plastic deformation resistance derived from the wheel tracking test and the dynamic modulus test differs in the size of the specimen and the test method, but the average of the two scores was applied since it is a test method describing the road environment due to cyclic load. The score for each item is 20 points, the maximum score is 100 points (total of 5 items) and decimal places are rounded up. The scores given to asphalt pavement materials (HMA, CRM 5%, CRM 5% + GNP 3%, CRM 5% + GNP 6%) in this study are relative scores to generally heated asphalt pavement. 

**Figure 14:**
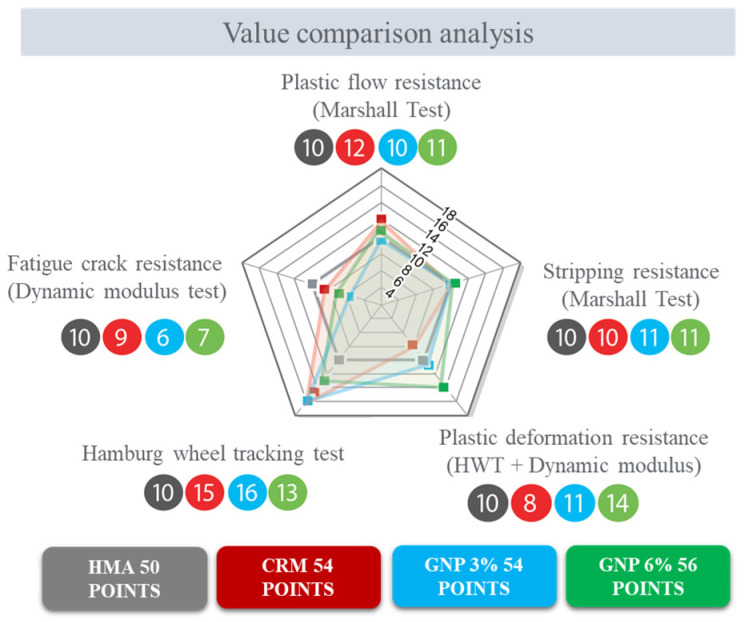
Results of performance evaluation analysis.

Figure 14 summarizes the result of the performance evaluation analysis. The controlled asphalt performance evaluation score was evaluated as 50 points, CRM 5% + GNP 6% asphalt pavement accounted for the highest result with 56 points, and CRM 5% and CRM 5% + GNP 3% asphalt pavement both achieved 54 points. As a result of the performance evaluation analysis, the characteristics of asphalt paving materials are as follows. Although the plastic deformation resistance of CRM (5%) asphalt pavement material was slightly low, it was confirmed that it could be suitably used as an anti-stripping agent as it showed much higher resistance than generally heated asphalt in the anti-stripping property. In the case of CRM (5%) + GNP (3%) asphalt paving materials, the resistance to anti-stripping accounted for the highest, but the resistance to fatigue cracking was lower than that of the reference asphalt mixture which is reflected in dynamic modulus test results. Among all testing mixtures, CRM (5%) + GNP (6%) asphalt pavement showed the highest performance among the four asphalt pavement materials. In particular, plastic deformation resistance was found to be the greatest, but resistance to fatigue cracking was determined to be relatively low. In conclusion, it was confirmed that the resistance to plastic deformation and anti-stripping of asphalt pavement materials using CRM is improved compared to general heated asphalt pavement materials. However, since the resistance to fatigue cracking can be somewhat reduced when CRM is mixed, it is suggested that an additional 6% GNP is necessary when applying to new pavement or expansion pavement construction. Therefore, in this study, we first proposed to apply it to small-scale emergency repair work.

### 3.4. Field Application

#### 3.4.1. Pothole Restoration 

**Figure 15:**
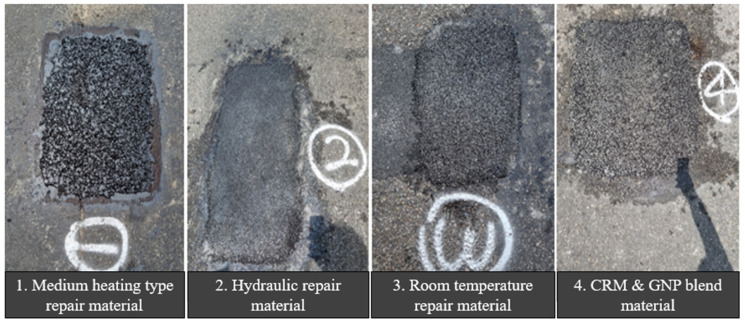
Appearance after construction by pavement material.

**Figure 16:**
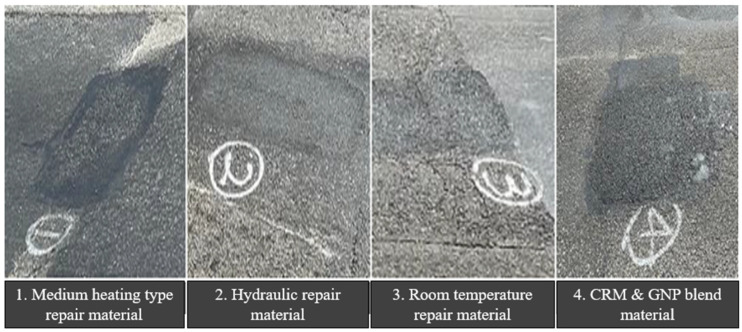
Result of visual inspection by compacting material after 10 days of construction.

Test construction was carried out in the summer rainy season (14 July 2021), and there was a possibility that potential damage may occur due to the heavy rain on 19 July based on the weather reports, so a visual inspection was conducted by visiting the site on 25 July, 10 days after construction. On 25 July 2021, a visual inspection was conducted to investigate the actual condition of the pavement. Figure 15 and Figure 16 illustrates the results of visual inspection by repair material. It was confirmed that all sections to which hydraulic-type repair materials, normal room temperature repair materials, and CRM (5%) asphalt pavement materials were applied were in good condition, and some irregularities occurred in the sections where medium-temperature type repair materials were used. 

The results of assessing the site porosity and compaction utilizing core samples taken from test pavement segments are demonstrated in Table 7. The proposed CRM_GNP mixtures and the popularly used commercial repairing asphalt mixtures both displayed almost equivalent levels of site compactness. Thus, employing the CRM GNP asphalt mixture for pothole restoration in pellets type is very practical for the actual application. Considering the field rutting measurement after 1 year (Table 8), the results indicate that the proposed asphalt pellet having CRM and GNP shows superior permanent deformation compared to the other methods. However, further long-term tracking has been conducted to govern its practical effectiveness. After a thorough assessment of the potential pothole countermeasures, Table 9 has been established to summarize the pros and cons of these solutions. Based on the suggestions and comments from Korean road and pavement specialists and in situ measurement, the CRM asphalt pellet approach represents a viable method for sustainable pavement rehabilitation and maintenance works, especially in small-scale damaged areas. 

In order to compare the unit cost of on-site heating method technology, the unit cost of heating technology for general heated asphalt pavement materials and the proposed asphalt pellet having CRM was compared. Considering the service life period of 6 years, the construction conditions are based on the daily construction volume of 100 m^2^, and reference is made to the construction standards. As a result of the comparison, the construction unit price per Peyong (3.3 m^2^) was 1,031,958 won for CRM packaging material heating technology while Conventional HMA heating technology requires at least 1,374,051 won. Table 10 shows the comparison of construction unit costs for each packaging material.

#### 3.4.2. FWDT Analysis

**Figure 17:**
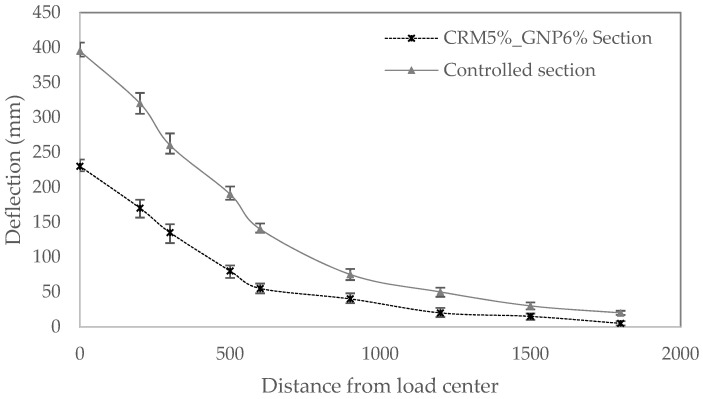
FWD method analysis: deflection data.

Figure 17 illustrates the FWD deflection based on the field construction sections. The field test result demonstrates that the displacement measurements for every segment vary significantly around the loading plate, and the difference between the deflection data from the two geophones diminishes as the distance from the loading plate increases, leading to convergence. As a result, the CRM section’s deflection at loading plate D0 was significantly lower than the controlled section, indicating a high settlement resilience. These findings suggest that the pavement structure of the CRM sections is superior.

**Figure 18:**
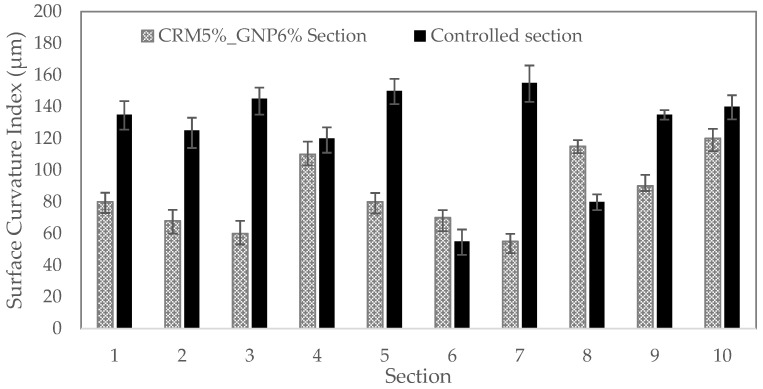
Layer index analysis (SCI).

The SCI value is compared in Figure 18 using displacement data. The structural integrity of the surface layer is represented by the SCI index. The structural condition rating is typically divided into two categories: severe zone for SCI > 400 and warning zone for SCI value from 200 to 400. Since all examined sites showed SCI values of less than 150, both options were regarded to have acceptable surface layer structural characteristics. Particularly, the SCI of the CRM sections has extremely low SCI values, suggesting that the layer’s features are rather superior to those of the conventional section.

### 3.5. Future Study

In the following research stages, additional long-term monitoring will be carried out to verify its operational efficacy. Additionally, a potential constraint to the viability of this CRM asphalt pellet pavement approach is the phase separation between CRM, CNP, and original asphalt binders. According to related research [18], phase separation can develop when the rubber particles are exposed to a heating source, causing a detrimental impact on the field performance of CRM pellet asphalt pavement. Hence, reducing phase separation is essential for the improvement of CRM pavement quality. As a result, in the following stage of this research, the phase integrity of CRM asphalt binder will be systematically studied in a series of laboratory tests to achieve an optimized CRM asphalt binder mix. 

Furthermore, there are significant regional and national differences in the CRM asphalt pellet storing circumstances (i.e., storage time and heating degree). Therefore, a secondary goal of the subsequent research was to evaluate multiple criteria of storage conditions from diverse locations. Considering the primary low-temperature cracking in asphalt pavements constructed in cold areas, the constrained pavement attempts to shrink as the temperature decreases, and the tensile strains increase to a dangerous threshold at which a fracture develops into critical cracking. Thus, proper investigation of the low-temperature behavior of CRM pellet asphalt pavement can provide comprehensive insights into the practical application of this method. In general, the proposed scopes for further study are expected to add value to the adaptability of this method, contributing to the optimized pavement restoration solution.

## 4. Conclusions

The objective of this study was to facilitate the application of pellet asphalt that contains Graphite Nanoplatelet (GNP) and Crumb Rubber Modifier (CRM) for road maintenance. The appropriate blending ratios of pellet-type asphalt paving materials were determined in this research by conducting laboratory tests on mixtures with CRM content ratios of 5%, 10%, and 20% and GNP content of 3% and 6%. The efficiency of the suggested technique and controlled combinations were compared using the Marshall stability test, Hamburg wheel tracking test, and dynamic modulus test. Afterward, a full-scale test bed was conducted by using popular commercial pothole restoration approaches. As a result of the performance evaluation analysis, the findings are presented as follows.

- The test results reveal that CRM content at 20% cultivates the noticeable drop in Mar-shall stability which may be inappropriate for pothole restoration purposes. Based on the general properties test, the CRM contents should be controlled at lower than 5% to ensure the standard strength.
- The test results confirmed that the CRM 5% mixture could be suitably used as an anti-stripping agent since it acquired higher resistance than general HMA. Regards this property, CRM (5%) + GNP (3%) asphalt concrete mixture obtained the highest re-sistance to stripping, but its fatigue cracking susceptibility was lower than that of the referenced mixture.
- The HWT test indicated that about 6 mm of plastic deformation occurred in the 5%CRM mixture and reference HMA. Meanwhile, the plastic deformation measured in the mixture of CRM (5%) + GNP (6%) is about 2.1 mm, whereas the mixture of CRM (5%) + GNP (3%) exhibits plastic deformation at approximately 3.3 mm. The dynamic stability of 5% CRM + 6% GNP mixes is considerably stronger than that of 5%CRM mix-tures (2423 times/mm compared to 1313 times/mm).
- The test findings show that CRM (5%) + GNP (6%) asphalt concrete mixture achieved the highest performance among the four conditions, suggesting its practical application to promote the environmentally friendly purpose. This can be attributed to the dense structure of HMA having proper content of GNP which results in the stronger bearing capacity and moisture resistance of the modified mixture. In particu-lar, the plastic deformation resistance of this condition represents the greatest value while the dynamic modulus of this condition shares the equivalent results compared to the controlled mixture.
- Based on the field test results, it was determined that all the repaired sections having a hydraulic type, normal room temperature type, and CRM (5%) + GNP (6%) asphalt pellet types exhibited excellent condition while medium-temperature type presented some abnormalities.
- Considering the availability of the material, the synthesized CNP graphite nano-flake powder can be easily provided by worldwide manufacturers while the CRM having a particle size of 30 mesh and organic disulfide activator is very popular in CRM production recently. Based on the utilization of by-product materials, the pro-posed asphalt pellet can save the pothole restoration cost by more than 30% compared to the conventional heated asphalt method.
- The levels of site compaction were comparable between the proposed CRM_GNP mixtures and the widely utilized commercially repairing asphalt mixtures. Therefore, using the CRM_GNP asphalt mixture in pellet form for pothole restoration is incredi-bly beneficial for actual implementation. Results demonstrate that the proposed as-phalt pellet containing CRM and GNP also exhibits superior permanent deformation when compared to the other approaches after one year. The Falling Weight Deflec-tometer test results also confirm that CRM pavement exhibits superior rutting re-sistance compared to that of the controlled section after 1 year of service life under en-vironmental and traffic load impacts.

According to the research’s findings, The CRM asphalt pellet approach is a feasi-ble solution for sustainable pavement maintenance and rehabilitation, particularly in small-scale damage areas such as potholes. In the following research stage, additional long-term monitoring has been carried out to verify its operational efficacy.

## Figures and Tables

**Table 1:** Aggregate and mineral filler properties.

Properties	Properties	Value
Aggregate	Relative apparent density [38]	2.68
	Water absorption [38]	0.17%
	Aggregate crushed value [39]	18.9%
	Los Angeles abrasion value [40]	26.1%
	Flakiness and elongation index [41]	13.1%
Mineral Filler	Relative apparent density [42]	2.29
	Moisture content [42]	0.07%

**Table 3:** Aggregate content by sieve size.

**Sieve Size** **(mm)**			25	20	13	10	5	2.5	0.6	0.3	0.15	0.08
**The Grain Size of Each Aggregate (%)**	**20 mm**											
	**13 mm**		100.0	100.0	97.6	79.8	17.4	5.4				
	**Crushed Sand**		100.0	100.0	100.0	100.0	98.4	75.6	39.2	23.4	12.1	5.3
	**Filler**		100.0	100.0	100.0	100.0	100.0	100.0	100.0	99.6	96.0	87.8
**Modified** **the Mixing Ratio of Each Aggregate (%)**	**20** **mm**	**-** **%**	0.0	0.0	0.0	0.0	0.0					
	**13** **mm**	**50.0%**	50.0	50.0	48.8	39.9	8.7	2.7				
	**Crushed Sand**	**47.0%**	47.0	47.0	47.0	47.0	46.2	35.5	18.4	11.0	5.7	2.5
	**Filler**	**3.0%**	3.0	3.0	3.0	3.0	3.0	3.0	3.0	3.0	2.9	2.6
**Synthetic Particle Size (%)**			100.0	100.0	98.8	89.9	57.9	41.2	21.4	14.0	8.6	5.1
**Median Particle Size (%)**			100.0	100.0	97.5	88.0	62.5	42.5	24.0	15.5	11.0	6.0
**Particle Size Range (%)**			100	100	95–100	84–92	55–70	35–50	18–30	10–21	6–16	4–8

**Table 4:** Material amount calculation.

Material		Weight by Asphalt Content (g)		
Type	Aggregate Remix (%)	6.0% (Rubber 5%)	6.5% (Rubber 10%)	7.0% (Rubber 20%)
20 mm	-			
13 mm	50.0	469	464	458
Crushed sand	47.0	440	436	431
Filler	3.0	28	28	27
Aggregate amount (g)	-	937.0	928.5	916.0
Rubber reforming	-	3.0	6.5	14.0
Amount of asphalt	-	60.0	65.0	70.0
(g, AP-5)	-	1000	1000	1000

**Table 5:** Ranges for road pavement structural condition grade.

	Percent Passing (%)				
Structural condition	Max deflection	RoC	SCI	BDI	BCI
Sound	<500	<100	<200	<100	<50
Warning	500 to 750	50 to 100	200 to 400	100 to 200	50 to 100
Severe	>750	>50	>400	>200	>100

**Table 6:** Derivation of optimum asphalt mixing ratio and Marshall stability by CRM content.

Division	Optimal Asphalt Mixing Ratio (OAC, %)	Porosity (%)	Marshall Stability (N)	Flow Value (1/10 mm)	Water Immersion Residual Stability (%)	Dynamic Stability (times/mm)
Standard	-	3–10	≥7350	20–40	≥75	≥750
CRM content 5%	6.0	3.5	12,726	31	94.8	1313
CRM content 10%	6.5	3.3	10.368	34	95.6	1189
CRM content 20%	7.0	2.6	8842	40	98.1	863

**Table 7:** Porosity and compactness of field test construction sections.

Classification	Measured Density (g/cm^3^)	Theoretical Density (g/cm^3^)	Field Pavement Porosity (%)	Field Compactness (%)
1. CRM and GNP blend material	2.239	2.389	5.9	97.4
2. Room temperature repair material	2.227	2.397	6.3	96.3
3. Hydraulic repair material	2.221	2.406	7.5	96.7
4. Medium heating type repair material	2.226	2.413	7.2	96.5

**Table 8:** The measurement result of transverse strain after 1 year.

	Rutting Measurement (mm)		
	Average	Max	Min
1. Medium heating type repair material	4.09	7.02	1.15
2. Hydraulic repair material	4.87	8.12	1.61
3. Room temperature repair material	5.04	7.24	2.83
4. CRM and GNP blend material	3.38	5.37	1.39

**Table 9:** Comparison of repair materials used in emergency repair work (KRW-Korea Currency).

Classification		1. Medium Heating Type Repair Material	2. Room Temperature Repair Material	3. Hydraulic Repair Material	4. CRM and GNP Blend Material
Price	25 kg/bag	10,000 KRW	10,500 KRW	20,000 KRW	-
	kg	400 KRW	420 KRW	800 KRW	150–300 KRW
Advantages		- Stored in a heating box at 70–80 °C at all times and repaired in a weakly heated state. - Easy adhesion and excellent durability compared to general emergency repair materials.	- Easy and quick repair in case of emergency.	- It reacts with water and hardens, so it can be installed even in the rainy season and rainy weather, and has better durability than general emergency repair materials.	- Compared to other repair materials, it is durable and has excellent moisture resistance. - Excellent economic feasibility and environmental efficiency by using waste rubber resources. - Possible to construct in rainy weather and winter.
Disadvantage		Requires a separate heating device	Re-breakage rate is high as durability decreases at a rapid rate.	Difficult to purchase in small quantities and difficult to manage temperature	Difficult to purchase in small quantities

**Table 10:** Comparison of construction unit cost by packaging material.

Classification (For Daily Construction Standards: 100 m^2^)	On-Site Asphalt Pellet Technology	Conventional HMA Heating Technology
Service life	6 years	
Unit price comparison (KRW/a)	1,031,958 (100%)	1,374,051 (133%)

## Data Availability

Data will be provided on request.

## References

[1] Wang X., Liu J., Wang Z., Jing H., Yang B. (2022). Investigations on Adhesion Characteristics between High-Content Rubberized Asphalt and Aggregates. Polymers.

[2] Qabur A., Baaj H., El-Hakim M. (2022). Incorporation of the Multi-Layer Plastic Packaging in the Asphalt Binders: Physical, Thermal, Rheological, and Storage Properties Evaluation. Polymers.

[3] Duan K., Wang C., Liu J., Song L., Chen Q., Chen Y. (2022). Research Progress and Performance Evaluation of Crumb-Rubber-Modified Asphalts and Their Mixtures. Constr. Build. Mater..

[4] Alsolieman H., Babalghaith A., Memon Z., Al-Suhaibani A., Milad A. (2021). Evaluation and Comparison of Mechanical Properties of Polymer-Modified Asphalt Mixtures. Polymers.

[5] Zhang J., Sesay T., You Q., Jing H. (2022). Maximizing the Application of RAP in Asphalt Concrete Pavements and Its Long-Term Performance: A Review. Polymers.

[6] Zhang X., Tang Z., Wang X., Wan C., Yang B. (2022). Performance Evaluation of Asphalt Binder and Mixture Modified by Pre-Treated Crumb Rubber. SSRN Electron. J..

[7] Chen X., Ma Z., Zhou J., Wang J., Zhang X., Zhao R., Tong J. (2022). Thermal Degradation Characteristics of Styrene-Butadiene-Styrene Copolymer Asphalt Binder Filled with an Inorganic Flame-Retarding Agent. Polymers.

[8] Wu W., Jiang W., Xiao J., Yuan D., Wang T., Xing C. (2022). Analysis of Thermal Susceptibility and Rheological Properties of Asphalt Binder Modified with Microwave Activated Crumb Rubber. J. Clean. Prod..

[9] Sahebzamani H., Zia Alavi M., Farzaneh O., Moniri A. (2022). Laboratory and Field Investigation of the Effect of Polymerized Pellets on the Fatigue and Low-Temperature Performance of Asphalt Mixtures. Constr. Build. Mater..

[10] Sahebzamani H., Alavi M.Z., Farzaneh O. (2018). Evaluating Effectiveness of Polymerized Pellets Mix Additives on Improving Asphalt Mix Properties. Constr. Build. Mater..

[11] Aarabi F., Batta R. (2020). Scheduling Spatially Distributed Jobs with Degradation: Application to Pothole Repair. Socio-Econ. Plan. Sci..

[12] Saputra R., Walvekar R., Khalid M., Mubarak N.M., Sillanpää M. (2021). Current Progress in Waste Tire Rubber Devulcanization. Chemosphere.

[13] Formela K. (2022). Waste Tire Rubber-Based Materials: Processing, Performance Properties and Development Strategies. Adv. Ind. Eng. Polym. Res..

[14] Boucher J., Friot D. (2017). Primary Microplastics in the Oceans. Mar. Environ. Res..

[15] Berendsohn R. (2018). Our Waste Tire Problem Is Getting Worse. Popular Mechanics.

[16] Jan Kole P., Löhr A.J., Van Belleghem F.G.A.J., Ragas A.M.J. (2017). Wear and Tear of Tyres: A Stealthy Source of Microplastics in the Environment. Int. J. Environ. Res. Public Health.

[17] Kordoghli S., Paraschiv M., Kuncser R., Tazerout M., Prisecaru M., Zagrouba F., Georgescu I. (2016). Managing the Environmental Hazards of Waste Tires. J. Eng. Stud. Res..

[18] Hakseo K., Soon-Jae L. (2013). Laboratory Investigation of Different Standards of Phase Separation in Crumb Rubber Modified Asphalt Binders. J. Mater. Civ. Eng..

[19] Zhang H., Zhang Y., Chen J., Liu W., Wang W. (2022). Effect of Activation Modes on the Property Characterization of Crumb Rubber Powder from Waste Tires and Performance Analysis of Activated Rubber-Modified Asphalt Binder. Polymers.

[20] Li Y., Abdelmagid A.A.A., Qiu Y., Yang E., Chen Y. (2022). Study on the Aging Mechanism and Microstructure Analysis of Rice-Husk-Ash-and Crumb-Rubber-Powder-Modified Asphalt. Polymers.

[21] Zhao Z., Wang L., Wang W., Shangguan X. (2022). Experimental Investigation of the High-Temperature Rheological and Aging Resistance Properties of Activated Crumb Rubber Powder/SBS Composite-Modified Asphalt. Polymers.

[22] Zhang H., Zhang Y., Chen J., Liu W., Wang W. (2022). Effect of Desulfurization Process Variables on the Properties of Crumb Rubber Modified Asphalt. Polymers.

[23] Liu W., Xu Y., Wang H., Shu B., Barbieri D.M., Norambuena-Contreras J. (2021). Enhanced Storage Stability and Rheological Properties of Asphalt Modified by Activated Waste Rubber Powder. Materials.

[24] Wu S., He R., Chen H., Li W., Li G. (2020). Rheological Properties of Sbs/Crp Composite Modified Asphalt under Different Aging Treatments. Materials.

[25] Nikol’skii V., Dudareva T., Krasotkina I., Gordeeva I., Gorbatova V., Vetcher A.A., Botin A. (2022). Mechanism of Multi-Stage Degradation in Hot Bitumen of Micronized Powder Elastomeric Modifiers from Worn-Out Tire’s Rubber. Polymers.

[26] Nikol’skii V., Dudareva T., Krasotkina I., Gordeeva I., Vetcher A.A., Botin A. (2022). Ultra-Dispersed Powders Produced by High-Temperature Shear-Induced Grinding of Worn-Out Tire and Products of Their Interaction with Hot Bitumen. Polymers.

[27] Li Y., Lyu Y., Xu M., Fan L., Zhang Y. (2019). Determination of Construction Temperatures of Crumb Rubber Modified Bitumen Mixture Based on CRMB Mastic. Materials.

[28] Xue Y., Zhao H., Wei X., Niu Y. (2019). Performance Analysis of Compound Rubber and Steel Slag Filler Modified Asphalt Composite. Materials.

[29] Wang T., Shi C., Yu Y., Xu G., Liu S., Wang H., Yang J., Gong M., Xu Y., Qie L. (2022). Mechanical Properties Evaluation of Crumb Rubber Asphalt Mixture for Elastic Trackbed. Constr. Build. Mater..

[30] Ghafari S., Ranjbar S., Ehsani M., Moghadas Nejad F., Paul P. (2023). Sustainable Crumb Rubber Modified Asphalt Mixtures Based on Low-Temperature Crack Propagation Characteristics Using the Response Surface Methodology. Theor. Appl. Fract. Mech..

[31] Li H., Cui C., Temitope A.A., Feng Z., Zhao G., Guo P. (2022). Effect of SBS and Crumb Rubber on Asphalt Modification: A Review of the Properties and Practical Application. J. Traffic Transp. Eng. (Engl. Ed.).

[32] Tan E.H., Zahran E.M.M., Tan S.J. (2022). The Optimal Use of Crumb Rubber in Hot-Mix Asphalt by Dry Process: A Laboratory Investigation Using Marshall Mix Design. Transp. Eng..

[33] Lyu L., Mikhailenko P., Piao Z., Fini E.H., Pei J., Poulikakos L.D. (2022). Unraveling the Modification Mechanisms of Waste Bio-Oils and Crumb Rubber on Asphalt Binder Based on Microscopy and Chemo-Rheology. Resour. Conserv. Recycl..

[34] Chen X., Ren D., Tian G., Xu J., Ali R., Ai C. (2023). Investigation on Moisture Damage Resistance of Asphalt Pavement in Salt and Acid Erosion Environments Based on Multi-Scale Analysis. Constr. Build. Mater..

[35] Wang C., Chen Q., Fu H., Chen J. (2018). Heat Conduction Effect of Steel Bridge Deck with Conductive Gussasphalt Concrete Pavement. Constr. Build. Mater..

[36] Renken P., Büchler S., Falchetto A.C., Wang D., Wistuba M.P. (2018). Warm Mix Asphalt-a German Case Study. Asph. Paving Technol. Assoc. Asph. Paving Technol. Tech. Sess..

[37] Li T., Jin Q., Jiang P., Sun H., Ding Y., Yan Z., Shi N. (2022). Performance Optimization of Modified Gussasphalt Binder Prepared Using Natural Asphalt. Front. Mater..

[38] (2004). Standard Test Method for Density, Relative Density (Specific Gravity), and Absorption of Coarse Aggregate.

[39] (2017). Standard Test Method for Determining the Percentage of Fractured Particles in Coarse Aggregate.

[40] (2014). Standard Test Method for Resistance to Degradation of Small-Size Coarse Aggregate by Abrasion and Impact in the Los Angeles Machine.

[41] (1999). Flat Particles, Elongated Particles, or Flat and Elongated Particles in Coarse Aggregate.

[42] (2001). Standard Specification for Mineral Filler For Bituminous Paving Mixtures.

[43] Song S., Liang M., Wang L., Li D., Guo M., Yan L., Zhang X., Ding W. (2022). Effects of Different Natural Factors on Rheological Properties of SBS Modified Asphalt. Materials.

[44] Xiao Z., Huang W., Wu K., Nie G., Hassan H.M.Z., Hu B. (2021). An Experimental Study on Properties of Pre-Coated Aggregates Grouting Asphalt Concrete for Bridge Deck Pavement. Materials.

[45] Gao L., Liu Y., Xie J., Yang Z. (2021). Cooling Performance and Thermal Radiation Model of Asphalt Mixture with Modified Infrared Powder. Materials.

[46] Jin J.H. (2021). A Study on the Characteristics of Low Carbon Mastic (Guss) Asphalt Concrete Pavement with a 20 °C Lower Transportation and Paving Temperature. J. Korean Asph. Inst..

[47] Al Mamun A. (2020). Development of Warm Mix Asphalt with the Aid of Microstructural Characterization.

[48] (2019). Standard Test Method for Penetration of Bituminous Materials.

[49] (2016). Standard Test Method for Softening Point of Bitumen (Ring-and-Ball Apparatus).

[50] (2008). Standard Test Method for Ductility of Asphalt Materials.

[51] (2009). Standard Test Method for Effects of Heat and Air on Asphaltic Materials (Thin-Film Oven Test).

[52] (2022). Standard Test Method for Effect of Heat and Air on a Moving Film of Asphalt (Rolling Rolling Thin-Film Oven Test).

[53] (2007). Accelerated Aging of Asphalt Binder Using a Pressurized Aging Vessel (PAV).

[54] (2011). Determining the Flexural Creep Stiffness of Asphalt Binder Using the Bending Beam Rheometer (BBR).

[55] Le J.L., Marasteanu M.O., Turos M. (2020). Mechanical and Compaction Properties of Graphite Nanoplatelet-Modified Asphalt Binders and Mixtures. Road Mater. Pavement Des..

[56] (2015). Standard Test Method for Marshall Stability and Flow of Asphalt Mixtures.

[57] (2017). Standard Method of Test for Hamburg Wheel-Track Testing of Compacted Hot Mixtures.

[58] (1995). Standard Test Method for Dynamic Modulus of Asphalt Mixtures 1.

[59] (2003). Standard Test Method for Deflections with a Falling Weight-Type Impulse Load Device.

[60] Dynatest FWD/HWD Test Systems (2007). Owner’s Man. Version 2.3.6.

[61] Lai J.C., Liu J., Huang C.W. (2020). The Application of Frequency-Temperature Superposition Principle for Back-Calculation of Falling Weight Deflectometer. Appl. Sci..

[62] Elseifi M.A., Abdel-Khalek A.M., Gaspard K., Zhang Z., Ismail S. (2012). Evaluation of Continuous Deflection Testing Using the Rolling Wheel Deflectometer in Louisiana. J. Transp. Eng..

[63] Horak E., Emery S., Maina J. Review of Falling Weight Deflectometer Deflection Benchmark Analysis on Roads and Airfields. Proceedings of the 11th Conference on Asphalt Pavements for Southern Africa: CAPSA15.

[64] Schnoor H., Horak E. Possible Method of Determining Structural Number for Flexible Pavements with the Falling Weight Deflectometer. Proceedings of the 31st Annual Southern African Transport Conference.

[65] Horak E., Hefer A., Maina J. Determining Pavement Number Values for Flexible Pavements Utilising Falling Weight Deflectometer Full Deflection Bowl Information. Proceedings of the Southern African Transport Conference.

[66] Horak E., Maree J.H., Van Wyk A.J. Procedures for Using Impulse Deflectometer (IDM) Measurements in the Structural Evaluation of Pavements. Proceedings of the Annual Transportation Convention.

[67] Horak E. Correlation Study of Falling Weight Deflectometer Determined Deflection Bowl Parameter and Surface Moduli. Proceedings of the Southern Africa Transportation Conference.

[68] Horak E. (2008). Benchmarking the Structural Condition of Flexible Pavements with Deflection Bowl Parameters. J. S. Afr. Inst. Civ. Eng..

